# Early limited nitrosamine exposures exacerbate high fat diet-mediated type 2 diabetes and neurodegeneration

**DOI:** 10.1186/1472-6823-10-4

**Published:** 2010-03-19

**Authors:** Ming Tong, Lisa Longato, Suzanne M de la Monte

**Affiliations:** 1 Department of Pathology (Neuropathology), Rhode Island Hospital, 593 Eddy Street, Providence, RI 02903, USA; 2 Department of Neurology, Rhode Island Hospital, 593 Eddy Street, Providence, RI 02903, USA; 3 Liver Research Center, Rhode Island Hospital, 55 Claverick Street, Providence, RI 02903, USA; 4 Pathobiology Program, Brown University, Box G, 222 Richmond Street, Providence, RI 02903, USA; 5 Warren Alpert Medical School of Brown University, Box G, 97 Waterman Street, Providence, RI 02912, USA

## Abstract

**Background:**

Type 2 diabetes mellitus (T2DM) and several types of neurodegeneration, including Alzheimer's, are linked to insulin-resistance, and chronic high dietary fat intake causes T2DM with mild neurodegeneration. Intra-cerebral Streptozotocin, a nitrosamine-related compound, causes neurodegeneration, whereas peripheral treatment causes DM.

**Hypothesis:**

Limited early exposures to nitrosamines that are widely present in the environment, enhance the deleterious effects of high fat intake in promoting T2DM and neurodegeneration.

**Methods:**

Long Evans rat pups were treated with N-nitrosodiethylamine (NDEA) by i.p. injection, and upon weaning, they were fed with high fat (60%; HFD) or low fat (5%; LFD) chow for 8 weeks. Cerebella were harvested to assess gene expression, and insulin and insulin-like growth factor (IGF) deficiency and resistance in the context of neurodegeneration.

**Results:**

HFD ± NDEA caused T2DM, neurodegeneration with impairments in brain insulin, insulin receptor, IGF-2 receptor, or insulin receptor substrate gene expression, and reduced expression of tau and choline acetyltransferase (ChAT), which are regulated by insulin and IGF-1. In addition, increased levels of 4-hydroxynonenal and nitrotyrosine were measured in cerebella of HFD ± NDEA treated rats, and overall, NDEA+HFD treatment reduced brain levels of Tau, phospho-GSK-3β (reflecting increased GSK-3β activity), glial fibrillary acidic protein, and ChAT to greater degrees than either treatment alone. Finally, pro-ceramide genes, examined because ceramides cause insulin resistance, oxidative stress, and neurodegeneration, were significantly up-regulated by HFD and/or NDEA exposure, but the highest levels were generally present in brains of HFD+NDEA treated rats.

**Conclusions:**

Early limited exposure to nitrosamines exacerbates the adverse effects of later chronic high dietary fat intake in promoting T2DM and neurodegeneration. The mechanism involves increased generation of ceramides and probably other toxic lipids in brain.

## Background

The prevalence rates of Alzheimer's Disease (AD), Parkinson's disease (PD), obesity, type 2 diabetes mellitus (T2DM), and metabolic syndrome have increased exponentially over the past several decades, and thus far show few hints of plateau [1-4]. The relatively short time interval flanking dramatic shifts in age-adjusted AD and PD mortality is consistent with exposure-related rather than genetic etiologies of these diseases. We have noted that the striking increases in AD and PD mortality rates followed sharply increased consumption of processed foods, use of preservatives, and demand for nitrogen-containing fertilizers [4]. A common theme resonating from these unnecessary lifestyle trends is that we have inadvertently increased our chronic exposures to nitrosamines (R1N(-R2)-N = O) and related compounds.

Nitrosamines form by chemical reactions between nitrites and secondary amines or proteins. Nitrosamines exert their toxic and mutagenic effects by alkylating N-7 of guanine, leading to increased DNA damage [5] and generation of reactive oxygen species such as superoxide (O_2_-) and hydrogen peroxide (H_2_O_2_). Consequences include increased lipid peroxidation, protein adduct formation, and pro-inflammatory cytokine activation [6]. Of note is that these very same molecular and biochemical pathogenic cascades are activated in major human insulin-resistance diseases, including T2DM, non-alcoholic steatohepatitis (NASH), and AD [7-13]. The concept that chronic injury caused by exposure to alkylating agents could result in malignancy and/or tissue degeneration is not far-fetched given the facts that: 1) chronic exposures to tobacco nitrosamines cause both lung cancer and emphysema; and 2) treatment with streptozotocin (STZ), a nitrosamine-related compound, causes hepatocellular carcinoma, pancreatic carcinoma, malignant brain tumors, T2DM, AD-type neurodegeneration, and/or hepatic steatosis, depending on dose and route of administration [14-22]. Therefore, although research on nitrosamine-related compounds has been focused on their mutagenic properties, thorough characterization of their non-neoplastic and degenerative effects is clearly warranted. In this regard, guidance may be obtained from what is already known about STZ-induced diseases.

STZ, like other N-nitroso compounds, causes cellular injury and disease by functioning as: 1) an alkylating agent and potent mutagen [14]; 2) an inducer of DNA adducts leading to increased apoptosis [23]; 3) a mediator of unscheduled DNA synthesis, triggering cell death [14]; 4) an inducer of single-strand DNA breaks and stimulus for nitric oxide (NO) formation following breakdown of its nitrosamine group [16]; and 5) an enhancer of the xanthine oxidase system leading to increased production of superoxide anion, H_2_O_2_, and OH^- ^radicals [24]. Ultimately, STZ-induced cellular injury, DNA damage, and oxidative stress cause mitochondrial dysfunction [16], ATP deficiency [25] and apoptosis. The structural similarities between STZ and nitrosamines, including N-nitrosodiethylamine (NDEA) and N-nitrosodimethylamine (NDMA) [26], together with experimental evidence that high doses of STZ cause cancer while lower doses cause diabetes or AD-type neurodegeneration with cognitive impairment [15,16,22] led us to hypothesize that while high doses of environmental and consumed nitrosamines cause cancer, lower, sub-mutagenic doses may promote insulin-resistance mediated degenerative diseases, including T2DM, AD, and possibly other neurological diseases.

Another key point is that obesity, T2DM, and cognitive impairment are likely inter-related, as suggested by the findings of: 1) increased risk for developing mild cognitive impairment (MCI), dementia, or AD in individuals with T2DM [7,27] or obesity/dyslipidemic disorders [28]; 2) progressive brain insulin resistance and insulin deficiency in AD [29-32]; 3) cognitive impairment in experimental animal models of T2DM and/or obesity [33,34]; 4) AD-type neurodegeneration and cognitive impairment in experimentally induced brain insulin resistance and insulin deficiency [21,22,35-37]; 5) improved cognitive performance in experimental models of AD [19] and in human subjects with AD or MCI after treatment with insulin sensitizer agents or intranasal insulin [38-44]; and 6) similar molecular, biochemical, and mechanistic abnormalities in T2DM and AD [7-13]. On the other hand, recent studies showed that HFD feeding causes obesity, T2DM, and cognitive impairment, but is not sufficient to cause AD [45,46]. Therefore, it's likely that chronic HFD feeding which results in peripheral insulin resistance may provide a second-hit, and that combined with low-dose nitrosamine or other environmental exposures, it may increase the severity of neurodegeneration.

In the present study, we examined the hypothesis that chronic HFD feeding and limited exposure to sub-mutagenic doses of NDEA function additively to cause insulin resistance with T2DM and neurodegeneration. We focused our investigations on the cerebellum because, apart from subcortical neurodegenerative diseases such as multiple system atrophy (MSA) [47-51], progressive supranuclear palsy (PSP) [52], cerebellar ataxias [48,53,54], PD [52,55], and motor neuron disease (MND) [56], in which cognitive impairment has been linked to cerebellar degeneration, there is growing evidence that cerebellar degeneration is also a feature of AD [57-59], Lewy body dementia (LBD) [60,61], and fronto-temporal dementia [62]. Although cerebellar degeneration in AD can be caused by superimposed PSP [63], MSA [64], or MND [65], it often occurs independently. Cerebellar degeneration in AD is manifested clinically by gait instability and increased falling [66], and structurally, by significant atrophy of the posterior cerebellar lobes, which correlates with cognitive impairment [67]. The major histopathological features of AD cerebellar atrophy include, reductions in Purkinje cell population, atrophy of the molecular and granule cell layers [68], increased amyloid deposition and gliosis in the cortex [69]; increased ubiquitin-immunoreactivity in senile plaques and degenerating neurites [70]; extensive abnormalities in dendritic spine density and synaptic structure in vestibulocerebellar, visual, and auditory pathways [71,72]; and degeneration of cerebellar white matter fibers with loss of climbing fibers and presynaptic varicosities [73]. In aggregate, synaptic pathology, rather than neurofibrillary tangles and plaques, represents the main correlate of cerebellar degeneration in AD.

In addition, cerebellar degeneration can be caused by systemic diseases such as diabetes mellitus [74], chronic alcoholism [75], or obesity with metabolic syndrome [45,46]. These systemic diseases share in common with primary central nervous system (CNS) degenerative diseases, impairments in cognition, and deficits in insulin and IGF signaling mechanisms, insulin/IGF responsive gene expression, and energy metabolism (glucose utilization) in the brain, including cerebellum [29,75-78]. Therefore, understanding the mechanisms by which lifestyle and environmental mediators promote neurodegeneration of the cerebellum has relevance to a broad spectrum of human CNS diseases.

## Methods

### Materials

Rabbit, mouse, or goat generated monoclonal or polyclonal antibodies to ubiquitin, tau, phospho-tau (AT8-S199, S202, T205), glial fibrillary acidic protein (GFAP), 4-hydroxy-2-nonenal (HNE), choline acetyltransferase (ChAT), amyloid precursor protein amyloid-β peptide (AβPP-Aβ), β-actin, were purchased from Chemicon (Tecumsula, CA), CalBiochem (Carlsbad, CA), or Molecular Probes (Eugene, OR). All other polyclonal and monoclonal antibodies and immunodetection reagents were purchased from Abcam (Cambridge, MA), Vector Laboratories (Burlingame, CA), Upstate (Billerica, MA), Chemicon (Temecula, CA), or Molecular Probes (Eugene, OR). The insulin ultra-sensitive ELISA kit was obtained from ALPCO Diagnostics (Salem, NH). Histochoice fixative was purchased from Amresco, Inc (Solon, OH). Antibodies to tumor necrosis factor-α (TNF-α) and interleukin-1β (IL-1β) were purchased from Invitrogen (Carlsbad, CA). The Free Fatty Acid Assay kit was purchased from Roche Applied Science (Indianapolis, IN). Antibodies to GSK3β, and phospho-GSK-3β (Ser9) were purchased from Cell Signaling (Danvers, MA). The OxyBlot assay for measuring protein carbonylation and the Milliplex-MAP Rat Adiponectin-Single Plex kit were purchased from Millipore Corp (Billerica, MA). The Amplex Red Cholesterol Assay Kit and Amplex UltraRed soluble fluorophore were purchased from Invitrogen (Carlsbad, CA). MaxiSorb 96-well plates used for ELISAs were from Nunc (Thermo Fisher Scientific; Rochester, NY). The MDA-586 kit to measure malondialdehyde was purchased from Oxis International, Inc. (Foster City, CA). The anti-IL6 and Leptin ELISA kits were from Peprotech (Rocky Hill, NJ). The TopCount NXT and ATP Lite assay kit were from Perkin-Elmer (Waltham, MA). Superblock-TBS, horseradish peroxidase conjugated antibodies, and SuperSignal Enhanced Chemiluminescence Reagent were from Pierce Chemical Co (Rockford, IL). QIAzol Lysis Reagent for RNA extraction and QuantiTect SYBR Green PCR Mix were obtained from Qiagen, Inc (Valencia, CA). The AMV 1^st ^Strand cDNA Synthesis kit was purchased from Roche Applied Science (Indianapolis, IN). The Serum Triglyceride Determination kit and synthetic oligonucleotides used in quantitative polymerase chain reaction (qPCR) assays were purchased from Sigma-Aldrich Co (St. Louis, MO). Fine chemicals were purchased from CalBiochem (Carlsbad, CA) or Sigma-Aldrich (St. Louis, MO).

### Experimental Model

Postnatal day 3 (P3) Long Evans rat pups (mean body weight 10 g) were given 3 alternate day intra-peritoneal (i.p.) injections of 20 μg NDEA or vehicle. Upon weaning, male rats (N = 8-10 per group) were pair-fed for 8 weeks with high fat (HFD) or low fat (LFD) chow diets. The HFD supplied 60% of the kcal in fat (54% from lard, 6% from soybean oil), 20% in carbohydrates, and 20% in protein, whereas the LFD supplied 10% of the kcal in fat (4.4% from lard, 5.6% from soybean oil), 70% in carbohydrates, and 20% in protein. Rats were weighed weekly, and food consumption was monitored daily. After an overnight fast (14 hours), rats were sacrificed by i.p. injection of pentobarbital (120 mg/kg). Blood and cerebella were harvested immediately. Blood or serum was used to measure glucose, insulin, cholesterol, triglycerides, and free fatty acid levels, as previously described [45,46]. Cerebella were harvested for histopathological, biochemical, and molecular studies. For histopathology, tissue samples were immersion fixed in Histochoice and embedded in paraffin. Histological sections (8-μm thick) were stained with Luxol Fast Blue, Hematoxylin, and Eosin (LHE) and examined under code. Adjacent sections were immunostained to detect glial fibrillary acidic protein (GFAP), ubiquitin, or 4-hydroxy-2-nonenal (HNE) as indices of neurodegeneration and oxidative stress as described elsewhere [79]. For molecular and biochemical assays, cerebella were snap-frozen in a dry ice-methanol bath and stored at -80°C. We studied cerebellar tissue because the cerebellum: 1) requires intact insulin/IGF signaling to maintain its structural and functional integrity [80,81]; 2) is severely damaged by i.c.-STZ mediated neurodegeneration [19,22]; 3) although relatively spared, it been shown to be a target of neurodegeneration in AD [57-59,82]; and 4) postmortem human studies demonstrated that AD cerebella exhibit insulin resistance [30]. Our experimental protocol was approved by the Institutional Animal Care and Use Committee at Lifespan-Rhode Island Hospital, and conforms to the guidelines set by the National Institutes of Health.

### Quantitative Reverse Transcriptase Polymerase Chain Reaction (qRT-PCR) Assays of Gene Expression

RNA was reverse transcribed using random oligodeoxynucleotide primers. The cDNAs of interest were amplified by PCR using gene specific primers (Table 1), and the products were detected with the Mastercycler ep realplex instrument and software (Eppendorf AG, Hamburg, Germany) [45,83]. Relative mRNA abundance was calculated from the ratios of specific mRNA to 18S measured in the same samples, and those data were used for inter-group statistical comparisons. Control studies included analysis of: 1) template-free reactions; 2) RNA that had not been reverse transcribed; 3) RNA samples pre-treated with DNAse I; 4) samples treated with RNAse A prior to the reverse transcriptase reaction; and 5) genomic DNA. All assays were performed in triplicate.

**Table 1:** Primer Pairs Used for Quantitative Reverse Transcriptase Polymerase Chain Reaction Assays

Primer	Direction	Sequence (5' → 3')	Position (mRNA)	Amplicon Size (bp)
Insulin	For	TTC TAC ACA CCC AAG TCC CGT C	145	135
Insulin	Rev	ATC CAC AAT GCC ACG CTT CTG C	279	
Insulin Receptor	For	TGA CAA TGA GGA ATG TGG GGA C	875	129
Insulin Receptor	Rev	GGG CAA ACT TTC TGA CAA TGA CTG	1003	
IGF-I	For	GAC CAA GGG GCT TTT ACT TCA AC	65	127
IGF-I	Rev	TTT GTA GGC TTC AGC GGA GCA C	191	
IGF-I Receptor	For	GAA GTC TGC GGT GGT GAT AAA GG	2138	113
IGF-I Receptor	Rev	TCT GGG CAC AAA GAT GGA GTT G	2250	
IGF-II	For	CCA AGA AGA AAG GAA GGG GAC C	763	95
IGF-II	Rev	GGC GGC TAT TGT TGT TCA CAG C	857	
IGF-II Receptor	For	TTG CTA TTG ACC TTA GTC CCT TGG	1066	91
IGF-II Receptor	Rev	AGA GTG AGA CCT TTG TGT CCC CAC	1156	
IRS-1	For	GAT ACC GAT GGC TTC TCA GAC G	604	134
IRS-1	Rev	TCG TTC TCA TAA TAC TCC AGG CG	737	
IRS-2	For	CAA CAT TGA CTT TGG TGA AGG GG	255	109
IRS-2	Rev	TGA AGC AGG ACT ACT GGC TGA GAG	263	
IRS-4	For	ACC TGA AGA TAA GGG GTC GTC TGC	2409	132
IRS-4	Rev	TGT GTG GGG TTT AGT GGT CTG G	2540	
ChAT	For	TCA CAG ATG CGT TTC ACA ACT ACC	478	106
ChAT	Rev	TGG GAC ACA ACA GCA ACC TTG	583	
AChE	For	TTC TCC CAC ACC TGT CCT CAT C	420	123
AChE	Rev	TTC ATA GAT ACC AAC ACG GTT CCC	542	
APP	For	GCA GAA TGG AAA ATG GGA GTC AG	278	199
APP	Rev	AAT CAC GAT GTG GGT GTG CGT C	476	
Tau	For	CGC CAG GAG TTT GAC ACA ATG	244	65
Tau	Rev	CCT TCT TGG TCT TGG AGC ATA GTG	308	
SPTLC-1	For	CTAACCTTGGGCAAATCGAA	2581	96
SPTLC-1	Rev	TGAGCAGGGAGAAGGGACTA	2676	
SPTLC-2	For	GGA CAG TGT GTG GCC TTT CT	1823	50
SPTLC-2	Rev	TCA CTG AAG TGT GGC TCC TG	1872	
CERS-1	For	TGC GTG AAC TGG AAG ACT TG	947	98
CERS-1	Rev	CTT CAC CAG GCC ATT CCT TA	1044	
CERS-2	For	CTC TGC TTC TCC TGG TTT GC	698	82
CERS-2	Rev	CCA GCA GGT AGT CGG AAG AG	779	
CERS-4	For	CGA GGC AGT TTC TGA AGG TC	1240	72
CERS-4	Rev	CCA TTG GTA ATG GCT GCT CT	1311	
CERS-5	For	GAC AGT CCC ATC CTC TGC AT	1254	92
CERS-5	Rev	GAG GTT GTT CGT GTG TGT GG	1345	
UGCG	For	GAT GCT TGC TGT TCA CTC CA	2682	67
UGCG	Rev	GCT GAG ATG GAA GCC ATA GG	2748	
SMPD-1	For	CAG TTC TTT GGC CAC ACT CA	1443	65
SMPD-1	Rev	CGG CTC AGA GTT TCC TCA TC	1507	
SMPD-3	For	TCT GCT GCC AAT GTT GTC TC	2704	98
SMPD-3	Rev	CCG AGC AAG GAG TCT AGG TG	2801	

*Abbreviations: IGF = insulin-like growth factor; IRS = insulin receptor substrate; ChAT = choline acetyltransferase; AChE = acetylcholinesterase; APP = amyloid precursor protein; SPTLC = serine palmitoyltransferase; CERS = ceramide synthase; UGCG = UDP-glucose ceramide glucosyltransferae; SMPD = sphingomyelin phosphodiesterase; bp = base pair.

### Enzyme-Linked Immunosorbant Assay (ELISA)

Tissue homogenates were prepared in radioimmunoprecipitation assay buffer containing protease and phosphatase inhibitors, as previously described [46]. Direct ELISAs were performed in 96-well Maxisorb plates [79]. In brief, proteins (40 ng/100 μl) adsorbed to well bottoms by over night incubation at 4°C, were blocked for 3 hours with 3% BSA in Tris buffered saline (TBS), then incubated with primary antibody (0.1-1.0 μg/ml) for 1 hour at room temperature. Immunoreactivity was detected with horseradish peroxidase (HRP)-conjugated secondary antibody (1:10000) and Amplex Red soluble fluorophore [79]. Amplex Red fluorescence was measured (Ex 579/Em 595) in a SpectraMax M5 microplate reader (Molecular Devices Corp., Sunnyvale, CA). Negative control reactions included substitutions with non-relevant primary or secondary antibodies, and omission of primary or secondary antibody. Immunoreactivities were normalized to protein content as determined with the NanoOrange Protein Quantification assay.

### Statistical Analysis

Data depicted in the graphs and tables represent the means ± S.E.M.'s for each group, with 8-10 samples included per group. Inter-group comparisons were made using one-way ANOVA and Bonferroni's multiple comparisons test for significance. Statistical analyses were performed using the GraphPad Prism 5 software (GraphPad Software, Inc., San Diego, CA). Software generated significant P-values are shown in the graphs or included in the tables.

## Results

### Effects of NDEA and HFD on Serum Biomarkers of T2DM (Table 2)

**Table 2:** High Fat Diet Feeding and NDEA Treatment Cause Type 2 Diabetes Mellitus

Assay	LFD+VEH	LFD+NDEA	HFD+VEH	HFD+NDEA	F-Ratio	P-Value
Body Wt (g)	265.100	266.600	315.300	308.400	3.42	0.04
	± 14.050	± 19.970	± 5.794	± 15.110		
Glucose (mg/dL)	111.5	128.8*	137.6***	150.8*** ξ	18.2	< 0.0001
	± 1.66	± 4.31	± 2.39	± 4.69		
Insulin (ng/ml)	0.0611	0.163*	0.324**	0.581*** ξ	22.69	< 0.0001
	± 0.017	± 0.038	± 0.074	± 0.060		
Leptin	4.649	4.775	11.49***^ξ^	15.73***^ξ^	37.22	< 0.0001
	± 0.789	± 0.386	± 0.678	± 1.473		
Adiponectin	20864	25195	16819 ξ	26635	3.36	0.048
	± 1454	± 3019	± 1254	± 1962		
Triglyceride (mg/ml)	0.399	0.424	0.173*** ξ	0.231*** ξ	38.2	< 0.0001
	± 0.028	± 0.005	± 0.011	± 0.032		
Free Fatty Acids (mM/mg prot)	0.150	0.114*	0.081*** ξ	0.063*** ξ	21.9	< 0.0001
	± 0.003	± 0.018	± 0.002	± 0.010		
Cholesterol (mg/ml)	0.943	0.986	0.552***^ξ^	0.748***^ξ^	6.53	0.004
	± 0.024	± 0.112	± 0.051	± 0.093		

Serum samples obtained at sacrifice and after an over-night fast were studied. Inter-group comparisons were made using ANOVA with Bonferroni's Multiple Comparison Tests. For all measurements, df = 3/36. Asterisks reflect significant differences relative to the LFD+ Vehicle-treated (VEH) control group (*P < 0.05; **P < 0.01; ***P < 0.001 relative to LFD+VEH). ξ = significant differences as follows: Glucose: P < 0.05 vs LFD+NDEA; Insulin: P < 0.001 vs LFD+NDEA; Adiponectin: P < 0.05 vs HFD+NDEA; Triglyceride: P < 0.001 vs LFD+NDEA; FFA: P < 0.001 vs LFD+NDEA; Cholesterol: P < 0.001 vs LFD+NDEA.

Mean body weights were higher in HFD fed relative to LFD fed rats. Mean fasting blood glucose and serum insulin concentrations were significantly lower in the LFD+VEH treated control group compared with all other groups. The mean levels of both serum glucose and insulin were next higher in the LFD+NDEA group, followed by the HFD group. The HFD+NDEA treated rats had the highest mean serum glucose and insulin levels. Correspondingly, serum leptin levels were significantly elevated in the HFD+VEH and HFD+NDEA treated relative to both LFD+VEH and LFD+NDEA groups. Serum adiponectin was lowest in the HFD+VEH group, and the difference relative to the HFD+NDEA treated group was statistically significant. Sera from rats in the HFD+VEH and HFD+NDEA treated groups had significantly lower mean triglyceride, free fatty acids, and cholesterol levels compared with LFD+VEH and LFD+NDEA treated groups. In addition, the serum free fatty acid level was significantly lower in the LFD+NDEA compared with LFD+VEH treated rats, whereas the triglyceride and cholesterol levels were similar in the two groups. Therefore, hyperglycemia, hyper-insulinemia, and hyper-leptinemia were features of chronic HFD feeding, and worsened by prior exposure to NDEA. In addition, early NDEA exposure alone was sufficient to cause mild but significant peripheral insulin resistance manifested by hyperglycemia and hyper-insulinemia in adults. In contrast, neither the NDEA exposure nor the chronic HFD feeding caused hyper-lapidarian, indicating that seemingly favorable serum lipid profiles can exist in the context of peripheral insulin resistance or T2DM. Similar results have been reported previously, in which the investigators generated models with much higher doses of NDEA [84]. One potential explanation for this paradox is that homeostatic mechanisms may have shifted toward increased storage of lipids/triglycerides in adipose tissue, skeletal muscle, and perhaps liver. Further studies will probe this hypothesis.

### Neuropathology of NDEA Exposure (Fig 1)

**Figure 1:**
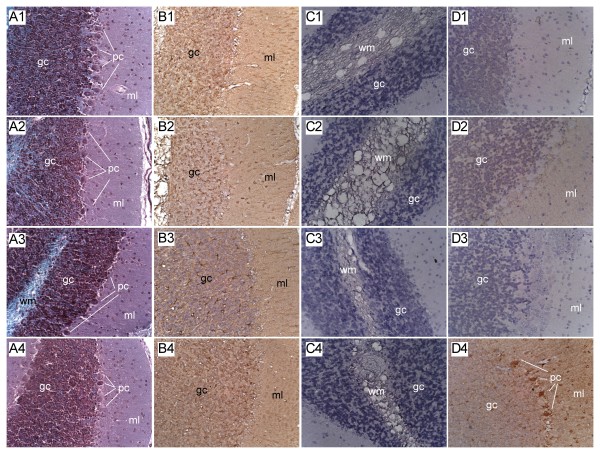
**Cerebellar Degeneration in NDEA, HFD-Fed and NDEA+HFD Treated Rats**. Long Evans rats were treated with 3 i.p. injections of vehicle or NDEA (N = 12/group) on alternate days beginning on P3. From P21 (weaning), rats were fed with high fat (60% of calories) or low fat (5% of calories) diets for 8 weeks, after which they were sacrificed to harvest cerebella for histopathological and immunohistochemical staining studies. Cerebella were preserved in Histofix and paraffin-embedded sections (8 microns) were stained with (A1-A4) Luxol fast blue, H&E. Adjacent sections were immunostained to detect (B1-B4) GFAP, (C1-C4) HNE, or (D1-D4) ubiquitin. Representative photomicrographs of cerebellar cortex from (A1-D1) LFD+vehicle treated controls, (A2-D2) LFD+NDEA treated, (A3-D3) HFD+vehicle treated, and (A4-D4) HFD+NDEA treated rats are shown. Immunohistochemical staining was performed using the ABC method, and revealed with DAB (brown precipitate)-see Experimental Procedures. Sections were lightly counterstained with Hematoxylin (blue) to help reveal the tissue architecture. Cerebellar layers: ml = molecular layer; pc = Purkinje cell layer; gc = granule cell layer; wm = white matter. Note focal pc loss in A2, and large zones of pc loss in A3 and A4. (Original Magnifications = 400×).

The LFD-fed, vehicle-treated control rats had cerebella with complex foliation, tri-laminar cortical architecture with richly populated granule and Purkinje cell layers, and uniform white matter cores (Fig. 1-A1). HFD feeding alone produced subtle changes characterized by focal loss of Purkinje neurons (Fig. 1-A3). NDEA exposure, with or without chronic HFD feeding, resulted in loss of Purkinje cells (Figs. 1-A2, 1-A4) and variable thinning of the granule cell layer. Immunohistochemical staining demonstrated similar levels and distributions of GFAP immunoreactivity in cells distributed in the granule layer of control (Fig 1-B1) and HFD-fed (Fig 1-B3) rats, but reduced labeling in the NDEA-treated group (Fig 1-B2), and increased GFAP immunoreactivity in the NDEA+HFD treated group (Fig 1-B4). Control and NDEA-treated rats had similarly very low levels of HNE immunoreactivity in white matter (Figs. 1-C1, 1-C2), whereas rats fed with HFDs, with or without NDEA exposure, had increased HNE immunoreactivity in white matter cores (Figs. 1-C3, 1-C4). In addition, the white matter was more vacuolated, suggestive of increased myelin degeneration, in these two groups. Ubiquitin immunoreactivity was virtually undetectable in control and NDEA-exposed cerebella (Figs. 1-D1, 1-D2), but slightly increased in the Purkinje and granule cell layers of HFD-fed cerebella (Fig. 1-D3). Rats exposed to NDEA, and also chronically fed with the HFD, had prominently increased ubiquitin immunoreactivity in Purkinje cells, and diffusely increased labeling of fine cell process in the granule and molecular layers of the cerebellar cortex (Fig. 1-D4). The increased ubiquitin and HNE immunoreactivities in both cortex and white matter suggest that oxidative injury associated with NDEA + HFD feeding occurred in neuronal and glial cell populations.

### NDEA-Treatment Plus Chronic HFD Feeding Causes Sustained Abnormalities in Gene Expression Including Impairments in Insulin/IGF Signaling Mechanisms in the Brain

We used qRT-PCR analysis to quantify long-term effects of NDEA ± HFD exposure on amyloid precursor protein (AβPP), Tau, choline acetyltransferase (ChAT), and acetylcholinesterase (AChE), i.e. molecular indices of neurodegeneration and cholinergic homeostasis. In addition, we measured gene expression corresponding to insulin and IGF polypeptides and receptors, and insulin receptor substrates (IRSs) that transmit signals required for growth, survival, energy metabolism, and neuronal plasticity downstream of the insulin and IGF receptors (Table 3). Early NDEA exposure alone was sufficient to significantly reduce the mean mRNA levels of AβPP (P < 0.05), Tau (P < 0.01), insulin receptor (P < 0.001), IGF-2 receptor (P < 0.01), and IRS-2 (P < 0.05) relative to control (LFD+VEH). Chronic HFD feeding significantly reduced ChAT (P < 0.05) and IRS-1 (P < 0.01) expression. HFD+NDEA reduced Tau (P < 0.01), ChAT (P < 0.05), and insulin (P < 0.05), but had no significant effect on the mean mRNA levels of AChE, IGF-1, IGF-2, IGF-1 receptor, or IRS-4. Therefore, it appears that the inhibitory effects of NDEA on insulin receptor, IGF-2 receptor, and IRS-2 were muted by the chronic HFD feeding. Moreover, the main effect of NDEA, irrespective of HFD feeding, was to reduce tau gene expression, whereas chronic HFD feeding, irrespective of NDEA treatment, significantly inhibited ChAT. The only unique effect of HFD+NDEA treatment was to reduce insulin gene expression in the brain.

**Table 3:** Effects of High Fat Diet and NDEA Exposure on Biomarkers of Insulin and IGF Resistance in the Cerebellum

mRNA	LFD+VEH	LFD+NDEA	HFD+VEH	HFD+NDEA	F-Ratio	P-Value
AβPP	7.007	3.129*^ξ^	8.960	6.796	4.918	0.006
	± 0.828	± 0.309	± 1.542	± 1.287		
Tau	12.230	6.586**^ξ^	11.230	7.284**^ξ^	6.612	0.001
	± 1.098	± 1.329	± 1.248	± 0.500		
AChE	2.829	4.171	3.719	3.562		
	± 0.178	± 0.698	± 0.618	± 0.412		
ChAT	0.701	0.780	0.519*	0.485*	3.524	0.020
	± 0.045	± 0.122	± 0.039	± 0.037		
Insulin	0.754	0.617	0.691	0.584*	3.065	0.040
	± 0.048	± 0.047	± 0.040	± 0.038		
IGF-1	0.957	0.717	0.558	0.630		
	± 0.119	± 0.179	± 0.063	± 0.060		
IGF-2	12.000	15.460	17.930	11.140		
	± 1.800	± 4.684	± 6.163	± 2.453		
Insulin R	17.090	7.59***^ξ^	16.590	16.170	9.824	< 0.001
	± 1.547	± 0.808	± 1.572	± 1.685		
IGF-1R	5.031	4.331	3.052	4.511		
	± 0.525	± 0.982	± 0.297	± 0.392		
IGF-2R	5.677	2.641** ξ	5.289	4.797	6.201	0.002
	± 0.548	± 0.432	± 0.693	± 0.466		
IRS-1	5.559	4.167	3.254**	5.276	4.532	0.009
	± 0.411	± 0.725	± 0.207	± 0.505		
IRS-2	7.701	4.834*	5.278	7.571	3.403	0.028
	± 0.509	± 0.934	± 0.286	± 1.198		
IRS-4	0.135	0.104	0.119	0.091		
	± 0.022	± 0.024	± 0.019	± 0.017		

Gene expression was measured by qRT-PCR with results normalized to 18S rRNA (see Methods). Values correspond to mRNA/18S × 10^-6^. Inter-group comparisons were made using ANOVA and the post-hoc Bonferroni multiple comparison test. For all measurements, df = 3/36. Asterisks (*P < 0.05; **P < 0.01; ***P < 0.001) reflect significant differences relative to LFD controls. ξ = significant differences as follows: AβPP: P < 0.05 vs HFD+VEH and HFD+NDEA; Tau: P < 0.01 vs HFD+VEH; ChAT: P < 0.05 vs LFD+NDEA; Insulin R: P < 0.001 vs HFD+VEH and HFD+NDEA; IGF-2R: P < 0.001 vs HFD+VEH and HFD vs NDEA; IRS-1: P < 0.01 vs HFD+NDEA and P < 0.05 vs LFD+NDEA.

### Effects of NDEA and Chronic HFD Feeding on Protein Biomarkers of Neurodegeneration (Fig 2)

**Figure 2:**
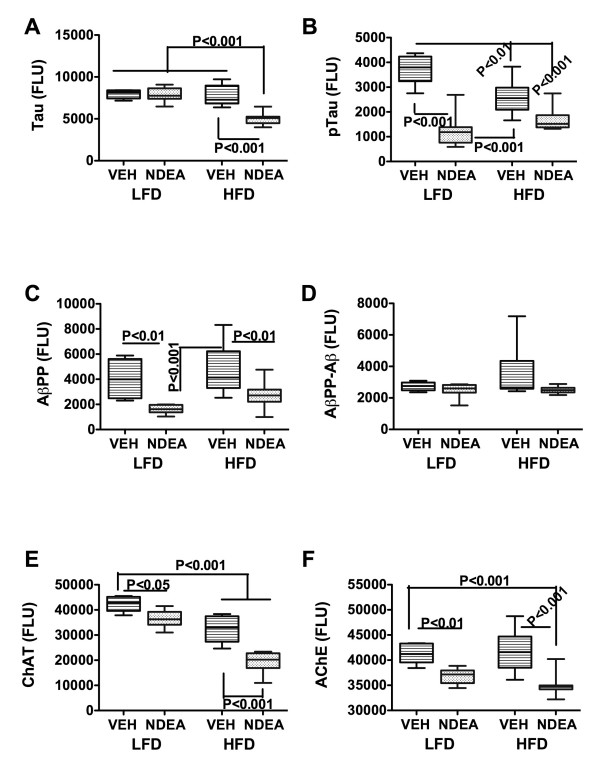
**Effect of HFD feeding on molecular indices of AD-type neurodegeneration**. Cerebellar protein homogenates were used to measure (A) Tau; (B) phospho (p)-Tau; (C) AβPP, (D) AβPP-Aβ, (E) choline acetyltransferase (ChAT); or (F) acetylcholinesterase (AChE) by direct binding ELISA. Immunoreactivity was detected with HRP-conjugated secondary antibody and Amplex Red soluble fluorophor. Fluorescence light units (FLU) were measured (Ex 579 nm/Em 595 nm) in a Spectromax M5, and results were normalized to sample protein content in the wells. Box plots depict mean, range ± S.D. of results (N = 8-10/group). Inter-group comparisons were made using ANOVA with the post-hoc Bonferroni multiple comparisons test of significance. Significant P-values are indicated within the panels.

ELISAs were used to measure sustained effects of NDEA treatment and/or chronic HFD feeding on Tau, phospho-Tau, AβPP, AβPP-Aβ, ChAT, and AChE levels in brain tissue. Early limited exposure to NDEA significantly reduced the mean levels of pTau (P < 0.001), AβPP (P < 0.01), ChAT (P < 0.05), and AChE (P < 0.01) relative to the levels measured in LFD+VEH treated controls. In addition, in the LFD+VEH group, the mean levels of pTau (P < 0.001), AβPP (P < 0001), and AChE (P < 0.05) were significantly lower than in the chronic HFD-fed group. Chronic HFD feeding alone (HFD+VEH) significantly reduced pTau (P < 0.01) and ChAT (P < 0.001) relative to control, whereas early NDEA exposure plus later chronic HFD feeding significantly reduced Tau (P < 0.001), pTau (P < 0.001), ChAT (P < 0.001), and AChE (P < 0.001) relative to control and chronic HFD fed rats. In addition, the mean levels of Tau (P < 0.001) and ChAT (P < 0.001) were significantly lower in the HFD+NDEA compared with the LFD+NDEA treated group. Therefore, the major adverse effects of chronic HFD feeding were to significantly reduce pTau and ChAT immunoreactivities relative to control, while early limited exposure to NDEA significantly reduced pTau, AβPP, ChAT, and AChE. Combined exposure to HFD and NDEA produced similar effects as NDEA-treatment alone with regard to the reductions in pTau, AβPP, and AChE, but had additive or synergistic effects with regard to reductions in Tau and ChAT immunoreactivity.

### Effects of NDEA and Chronic HFD Feeding on Protein Biomarkers of Oxidative Injury and Inflammation (Fig 3)

**Figure 3:**
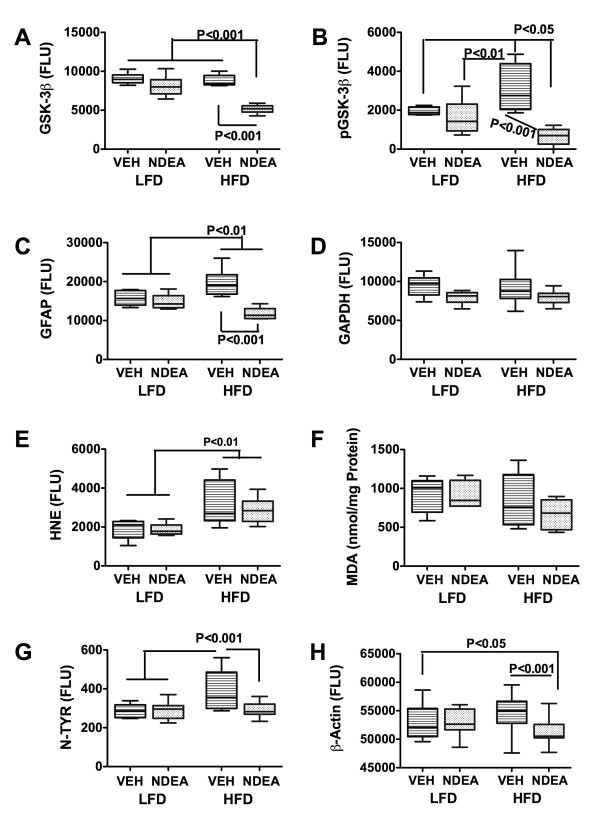
**Effect of HFD feeding on molecular indices of oxidative stress and neurodegeneration**. Cerebellar protein homogenates were used to measure (A) GSK-3β; (B) phospho (p)-GSK-3β; (C) GFAP; (D) GAPDH; (E) HNE; (F) malondialdehyde, MDA; (G) Nitrotyrosine, N-TYR; or (H) β-Actin; by direct binding ELISA. Immunoreactivity was detected with HRP-conjugated secondary antibody and Amplex Red soluble fluorophor. Fluorescence light units (FLU) were measured (Ex 579 nm/Em 595 nm) in a Spectromax M5, and results were normalized to sample protein content in the wells. Box plots depict mean, range ± S.D. of results (N = 8-10/group). Inter-group comparisons were made using ANOVA with the post-hoc Bonferroni multiple comparisons test of significance. Significant P-values are indicated within the panels.

ELISAs were used to measure sustained effects of early limited NDEA treatment and/or chronic HFD feeding on glycogen synthase kinase 3β (GSK-3β), pGSK-3β (Ser 9), GFAP, glyceraldehydes-3-phosphate dehydrogenase (GAPDH), HNE, Nytrotyrosine, (N-Tyr), β-Actin, IL-1β, TNF-α, and IL-6 immunoreactivities. In addition, we measured MDA and protein carbonylation levels in brain. The objective was to assess oxidative stress and biomarkers of injury. Early limited exposure to NDEA had no significant effect on any of the indices measured relative to control. Chronic HFD feeding significantly increased the mean levels of pGSK-3β, GFAP, and N-Tyr relative to all other groups (P < 0.05 or better). Combined early limited NDEA exposure plus later chronic HFD feeding significantly reduced the mean levels of GSK-3β and GFAP relative to all other groups (P < 0.01 or better), and pGSK-3β and β-Actin relative to the LFD+VEH control (both P < 0.05) and HFD+VEH (both P < 0.001) treated groups. The finding of proportionally greater reductions in pGSK-3β relative to total GSK-3β in the HFD+NDEA group indicates that the combined treatments caused a net increase in activated GSK-3β. The mean levels of HNE were similar in the HFD+VEH and HFD+NDEA groups, and both were significantly higher than in the LFD+VEH and LFD+NDEA groups (P < 0.01). We did not detect significant inter-group differences with respect to MDA (Fig 3), protein carbonylation, or IL-1β, TNF-α, and IL-6 immunoreactivities (data not shown).

### NDEA-Mediated Increases in Pro-Ceramide Gene Expression

**Figure 4:**
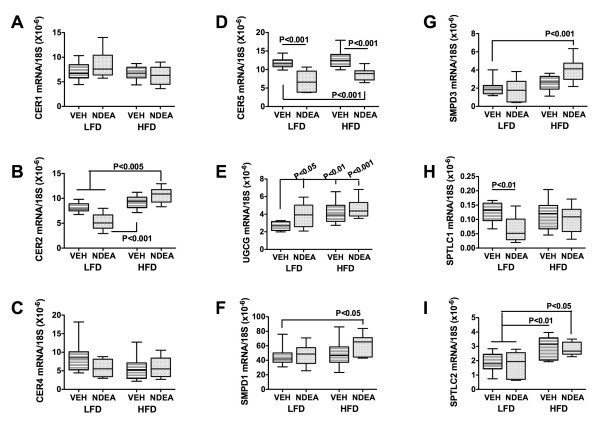
**Effect of HFD feeding on pro-ceramide gene expression in brain**. Total RNA extracted from cerebellar tissue was reverse transcribed using random oligodeoxynucleotide primers, and the resulting cDNA templates were used in qRT-PCR assays to measure (A) Ceramide synthase (CER)1, (B) CER2, (C) CER4, (D) CER5, (E) UDP-glucose ceramide glycoysltransferase (UGCG), (F) sphingomyelin phosphodiesterase 1 (SMPD1), (G) SMPD3, (H) Serine palmitoyltransferase 1 (SPTLC1), and (I) SPTLC2. The mRNA levels were normalized to 18S rRNA measured in the same templates. Graphs depict the mean, range ± S.D. of gene expression levels (N = 8 per group). Inter-group comparisons were made using ANOVA with the post-hoc Bonferroni multiple comparisons test of significance. Significant P-values are indicated within the panels.

We investigated the potential role of pro-ceramide genes as mediators of NDEA ± HFD associated neurodegeneration because ceramides: 1) can be generated in brain [85-88]; 2) cause cytotoxicity and insulin resistance [83,88]; and 3) are increased in brains with neurodegeneration [85,89-91]. We measured mRNA levels of ceramide synthases (CER), UDP glucose ceramide glycosyltransferase (UGCG), serine palmitoyltransferase (SPTLC), and sphingomyelin phosphodiesterases (SMPD), due to their demonstrated relevance to neurodegeneration [45,83]. HFD feeding alone increased the mean levels of UGCG (P < 0.01) and SPTLC2 (P < 0.01), whereas NDEA treatment significantly reduced CER2 (P < 0005), CER5 (P < 0.001), and SPTLC1 (P < 0.01), but increased UGCG (P < 0.05) mRNA levels (Fig 4). HFD+NDEA feeding significantly increased CER2 (P < 0.005), UGCG (P < 0.001), SMPD1 (P < 0.05), SMPD3 (P < 0.001), and SPTLC2 (P < 0.05), but decreased CER5 (P < 0.001) relative to control. The unique effects of HFD+NDEA feeding compared with either treatment alone were to significantly increase CER2, SMPD1 and SMPD3 gene expression (Fig 4). NDEA and/or chronic HFD feeding did not significantly alter CER1 or CER4 gene expression.

## Discussion

Our studies examined the degree to which limited early life NDEA exposure exacerbates the effects of chronic HFD feeding on the development of T2DM and neurodegeneration. The importance of this work is that morbidity and mortality rates from T2DM and neurodegeneration have soared over the past several decades, suggesting that exposures rather than genetics dictate their etiologies. Our over-arching hypothesis is that shifts in lifestyles and economics have led us to chronically consume excess fat, and get exposed to agents that cause insulin resistance. Consideration given to potential pathogenic agents was focused by the experimental evidence showing that STZ, a nitrosamine-related chemical, is mutagenic in high doses [14], but causes insulin-resistance diseases at lower levels or more limited durations of exposure, including early in life [15-22,92]. While STZ is generally not available to consumers, nitrosamines and related compounds are widely present in our environment and contaminate food sources. Therefore, we entertained the hypothesis that either limited or chronic low-level exposures to nitrosamines account for the observed shifts in morbidity and mortality from insulin resistance diseases. Moreover, given the clear role of high dietary fat intake as a mediator of obesity, T2DM, or cognitive impairment, we proposed that the combined effects of HFD and NDEA exposure may act additively or synergistically to cause insulin resistance diseases, including T2DM and neurodegeneration.

We generated an in vivo model in which rat pups were treated with NDEA at doses that were 5- to 500-fold lower than the cumulative doses needed to produce cancer in experimental animals [93-96], and beginning in early adolescence, we pair-fed the rats with either high (60%) or low (5%) fat containing diets. The NDEA doses were selected to be far below those needed for carcinogenesis and were based on empirical studies demonstrating absence of acute toxic effects in the rats. Longer durations of NDEA exposure were not used in order to avoid the possibility that the cumulative doses would lead to malignant transformation. The use of young rats enabled us to compare results with previous observations in the STZ model [81]. Moreover, longitudinal studies of nuns revealed that neuro-cognitive deficits precede the onset of dementia by decades [97,98], suggesting that early life exposures may contribute to the pathogenesis of AD, perhaps through gene imprinting. Although chronic HFD feeding and limited NDEA exposure increased body weight and caused T2DM/peripheral insulin resistance with fasting hyperglycemia, hyper-insulinemia, and hyper-leptinemia [99,100], the rats were not obese and they did not have hyper-lipidemia. Instead, the serum lipid profile was favorable, consistent with a previous report of a related experimental model [84].

We focused our investigations on the cerebellum because the cerebellum can be adversely affected in AD, as well as other neurodegenerative diseases [47,50,53,56,59,60,65,68,69,71], and cerebellar degeneration causes cognitive impairment [49,57-59,62,63,66,67,72]. Previous studies demonstrated significant structural, functional, and metabolic abnormalities in AD cerebella [57-59,82], including insulin and IGF resistance [30], similar to the findings in more traditional targets of AD, i.e. cortical and limbic structures. Finally, by focusing on one brain region, we were able to investigate a novel concept concerning potential additive or synergistic effects of double-hits, i.e. nitrosamine plus HFD exposures, on neurodegeneration in general.

Although both NDEA and HFD feeding caused structural injury and impaired gene expression in ways that could cause insulin/IGF resistance in the brain, their specific effects were not identical. The main effect of NDEA, with or without HFD feeding, was to reduce mRNA levels of insulin receptor, IGF-2 receptor, and IRS-2, which would have impaired signaling at the receptor level, and downstream through IRS-2, one of main docking proteins responsible for transmitting survival, growth, metabolism, and plasticity pathway signals [80,81]. Correspondingly, NDEA treatment inhibited tau and ChAT, which are regulated by insulin and IGF [81], and are reduced in brains with AD [81]. Reductions in tau, a major structural protein in CNS neurons, could result in cytoskeletal collapse and synaptic disconnection. Alternatively, the finding could reflect neuronal loss associated with neurodegeneration. The reduced levels of ChAT reflect deficits in acetylcholine homeostasis that contribute to cognitive impairment with neurodegeneration [101,102]. Correspondingly, in preliminary studies, we detected evidence of significant spatial learning and memory deficits in NDEA and NDEA+HFD treated rats by Morris Water Maze testing (Additional file 1, Table S1). The potential contribution of non-cerebral, specifically cerebellar degeneration to cognitive impairment was demonstrated in human subjects with PD, whereby mild cognitive impairment was found to be associated with reduced nicotinic acetylcholine receptor binding in the cerebellum and brainstem [103].

In previous studies using a mouse model of diet-induced obesity [45,46], we showed that chronic HFD feeding causes brain insulin resistance [46]. Similarly, herein we demonstrated that the HFD-fed rats had reduced levels of brain IRS-1 mRNA, which would have been sufficient to cause brain insulin resistance due to impaired transmission of insulin receptor activated signals; correspondingly, ChAT expression was reduced. In addition, HFD feeding increased GFAP, HNE, and N-Tyr immunoreactivities, consistent with findings in previous studies [45,46], and providing further evidence that HFD feeding contributes to neurodegeneration by increasing gliosis/astrocyte activation and oxidative stress with lipid peroxidation, as occur in AD. The finding that chronic HFD feeding did not significantly alter tau or AβPP expression also supports our previous conclusion that HFD feeding contributes to, but is not sufficient to cause AD-type neurodegeneration [45,46].

The combined effect of early, limited NDEA exposure plus chronic HFD feeding significantly reduced insulin and ChAT mRNA levels, and ChAT, AChE, GSK-3β, and pGSK-3β immunoreactivities. In addition, tau and ChAT expression levels were lowest in the HFD+NDEA group. Since tau and ChAT are regulated by insulin/IGF signaling [81], the impairment in insulin gene expression was likely pivotal in mediating these effects of HFD+NDEA exposure. In the HFD+NDEA group, both GSK-3β and pGSK-3β levels were reduced, but relative reductions were greater for pGSK-3β compared with total GSK-3β, reflecting a net relative increased in GSK-3β activity, similar to the findings in AD and PD in humans [29,30,104]. Therefore, HFD+NDEA treated rats exhibited persistent brain oxidative stress associated with increased levels of HNE (lipid peroxidation), N-Tyr, and GSK-3β activity. Although the NDEA exposure was delivered within a brief window during the human equivalent of childhood or early adolescence, its adverse effects on the brain were sustained into adulthood, similar to the findings with respect to the i.c.-STZ model [19,22]. Conceivably, such prolonged and possibly progressive deficits may occur because nitrosamines promote formation of DNA and protein adducts [105-107] that can serve as persistent sources of oxidative stress, and cause further DNA damage and protein dysfunction.

Recently, we demonstrated a role for ceramide-mediated neurodegeneration in a model of diet-induced obesity with T2DM [45], and showed that in vitro ceramide exposure causes neurodegeneration with impairments in neuronal viability, energy metabolism and insulin/IGF signaling mechanisms [83], consistent with previous reports [45,83,85,88-90,108-110]. Moreover, other investigators have demonstrated significantly increased levels of ceramides and other sphingolipids in brains with neurodegeneration [76,90,110-114]. Therefore, we measured pro-ceramide mRNA levels to determine if the HFD or NDEA-associated neurodegeneration and insulin/IGF resistance were likely mediated by increased brain ceramide levels. Those studies demonstrated strikingly increased expression of several genes regulating ceramide production via both de novo biosynthesis or sphingomyelin degradation pathways in NDEA-treated rats, irrespective of chronic HFD feeding. Since NDEA is lipid soluble [115,116] and can cross the blood-brain barrier [117], it is probable that the neurodegeneration with insulin/IGF resistance associated with limited NDEA exposure was mediated by direct and persistent neurotoxic effects of compound.

## Conclusions

In conclusion, we have demonstrated that limited early exposure to sub-mutagenic doses of NDEA causes T2DM and neurodegeneration with impairments in insulin/IGF signaling mechanisms, and deficits in cholinergic and neuronal cytoskeletal gene and protein expression in brain, whereas chronic HFD feeding alone produces more restrictive deficits in insulin/IGF signaling mechanisms with reduced ChAT expression and increased oxidative stress. The combined exposures caused overlapping structural and molecular abnormalities that were additive with regard to the breadth of insulin/IGF signaling molecules that were impaired, and synergistic with respect to the degrees in which ChAT and tau expression were reduced and pro-ceramide genes increased. Since experimentally, exposure to neurotoxic ceramides produces similar effects, their increased levels in cerebrospinal fluid could serve as biomarkers of insulin-resistance mediated neurodegeneration. Finally, the findings suggest that our insulin resistance disease epidemics are linked to sub-mutagenic exposures to nitrosamines and related compounds, combined with chronic consumption of high fat content foods, indicating that these diseases are preventable.

## Abbreviations

AβPP: amyloid-β-precursor protein; AβPP-Aβ: amyloid-β peptide; AChE: acetylcholinesterase; AD: Alzheimer's disease; CER: Ceramide synthase; ChAT: choline acetyltransferase; ELISA: enzyme-linked immunosorbant assay; GFAP: glial fibrillary acidic protein; GSK-3β: glycogen synthase kinase-3β; H&E: hematoxylin and eosin; HFD: high fat diet; HNE: 4-hydroxy-2-nonenal; HRP: horseradish peroxidase; i.p.: intraperitoneal; IGF: Insulin like growth factor; IRS: Insulin receptor substrate; LFD: low fat diet; LHE: Luxol fast blue hematoxylin and eosin; MCE: mild cognitive impairment; NASH: non-alcoholic steatohepatitis; NDEA: N-nitrosodiethylamine; NDMA: N-nitrosodimethylamine; P3: postnatal day 3; qRT-PCR: quantitative reverse transcriptase polymerase chain reaction; SMPD: sphingomyelin phosphodiesterase; SPTLC: Serine palmitoyltransferase; STZ: Streptozotocin; T2DM: Type 2 diabetes mellitus; TBS: Tris buffered saline; UGCG: UDP-glucose ceramide glycoysltransferase.

## Competing interests

The authors declare that they have no competing interests.

## Authors' contributions

MT participated in the experimental design and the molecular and biochemical studies. LL contributed to the molecular and biochemical studies and participated in the data analysis. SMdlM conceived of the study, participated in the study design and coordination, performed statistical analysis, and drafted the manuscript. All authors read and approved the final manuscript.

## Pre-publication history

The pre-publication history for this paper can be accessed here:

http://www.biomedcentral.com/1472-6823/10/4/prepub

## Supplementary Material

Additional file 1 **Effects of HFD and NDEA on Learning: Morris Water Maze Test**. This supplementary table provides data showing adverse effects of NDEA exposure, chronic HFD feeding or both treatments on spatial learning and memory using the Morris Water Maze test. Click here for file
