# Supplementary material for: Early limited nitrosamine exposures exacerbate high fat diet-mediated type 2 diabetes and neurodegeneration
Source: BMC Endocr Disord. 2010 Mar 19;10:4. doi: 10.1186/1472-6823-10-4 (PMC3161394; doi:10.1186/1472-6823-10-4)
Supplement: Additional file 1 — Effects of HFD and NDEA on Learning: Morris Water Maze Test. This supplementary table provides data showing adverse effects of NDEA exposure, chronic HFD feeding or both treatments on spatial learning and memory using the Morris Water Maze test. [file 1472-6823-10-4-S1.DOC]

Table S1: Effects of HFD and NDEA on Learning-Morris Water Maze Test

|  | LFD | LFD+NDEA | HFD | HFD+NDEA | P-Value |
| --- | --- | --- | --- | --- | --- |
| Day 1 | 53.53 | 131.8** | 85.88 | 148.7** | 0.003 |
|  | ±4.075 | ±19.15 | ±5.842 | ±17.08 |  |
|  |  |  |  |  |  |
| Day 2 | 39.36 | 66.81 | 99.94* | 143.0*** | 0.0007 |
|  | ±5.059 | ±16.70 | ±13.39 | ±21.18 |  |

HFD and NDEA Treatments Impair Spatial Learning: Long Evans rats treated with vehicle or NDEA by i.p. injection, and subsequently fed with low-fat (LFD) or high fat (HFD) chow diets for 8 weeks. Rats (N=8 per group) were subjected to Morris Water Maze testing at 4 weeks of age. On Day 1 of testing, the platform was visible, but on Day 2, the platform was submerged. On each testing day, rats were given 3 trials, with a maximum of 120 seconds allowed to land on the platform, beyond which they were guided. Area under curve corresponding to latency required to locate and land on the platform was calculated for each series of trials each day. Data reflect the mean ± S.E.M. of AUC latencies on each day of testing. Data were analyzed using the Kruskal-Wallis one-way ANOVA and Dunn’s multiple comparison post-hoc test for significance. Significant P-values relative to control (LFD) are indicated by asterisks: *P<0.05; **P<0.01, ***P<0.001. Other inter-group differences were not statistically significant.
